# Suprarenal Extract in Heart Disease

**Published:** 1901-07

**Authors:** 


### Suprarenal Extract in Heart Disease.

Dr. Samuel Floersheim, of New York, has obtained marvelously good results from the use of suprarenal capsule in diseases of the heart. He uses the dried and powdered gland obtained from the sheep, the usual dose of which is three grains, to be packed loosely in a capsule and dissolved in the mouth. The full effect of the extract absorbed from the mouth is obtained in from five to ten minutes and lasts from a few minutes to three or more hours. Its administration is free from danger, there being no poisonous or cumulative effect. It is indicated in every form of cardiac disease and has no effect upon the normal heart. Dr. Floersheim's conclusions, based upon the results obtained in one hundred cases of heart disease, are as follows: "After administration of the suprarenal powder (1) a weak and irregular acting heart became stronger and more regular. (2) Dilated heart was contracted. (3) A diffused apex beat became localized. (4) The normal cardiac sounds, when indistinct, became clearer and more easily distinguished. (5) Patients who were very weak, with organic heart disease were improved. (6) No effect was observed in organic heart disease when the pulse was strong and regular.—*New York Medical Journal.*

